# Identification of a Novel Indel Variant in the *DARS2* Gene in Russian Patients with Leukoencephalopathy with Brainstem and Spinal Cord Involvement and Lactate Elevation

**DOI:** 10.3390/genes15050615

**Published:** 2024-05-11

**Authors:** Fatima M. Bostanova, Polina G. Tsygankova, Elena A. Larshina, Ilya O. Nagornov, Yulia V. Evseeva, Irina L. Krutikhina, Marina E. Dzhentemirova, Marina N. Kashlakova, Marina S. Petukhova, Inna V. Sharkova, Ekaterina Y. Zakharova

**Affiliations:** 1 Research Centre for Medical Genetics, 1 Moskvorechye St., 115522 Moscow, Russia; bostanova@med-gen.ru (F.M.B.);; 2 Ekaterinburg Research Institute of Viral Infections, 620030 Yekaterinburg, Russia; 3 Kirov Regional Children’s Clinical Hospital, 610050 Kirov, Russia; 4 State Budgetary Healthcare Institution “Magadan Region Center for Maternal and Child Health”, 685000 Magadan, Russia; 5 St. Petersburg State Budgetary Institution, City Polyclinic, No. 114, 197374 St. Petersburg, Russia

**Keywords:** leukoencephalopathy with brainstem and spinal cord involvement and lactate elevation (LBSL), the *DARS2* gene, white matter disorder, targeted gene sequencing, next-generation sequencing (NGS)

## Abstract

Background: Leukoencephalopathy with brainstem and spinal cord involvement and lactate elevation is an inherited disease caused by pathogenic biallelic variants in the gene *DARS2*, which encodes mitochondrial aspartyl-tRNA synthetase. This disease is characterized by slowly progressive spastic gait, cerebellar symptoms, and leukoencephalopathy with brainstem and spinal cord involvement. Case Presentation: Peripheral blood samples were collected from four patients from four unrelated families to extract genomic DNA. All patients underwent partial exon analysis of the *DARS2* gene using Sanger sequencing, which detected the c.228-21_228-20delinsC variant in a heterozygous state. Further DNA from three patients was analyzed using a next-generation sequencing-based custom AmpliSeq™ panel for 59 genes associated with leukodystrophies, and one of the patients underwent whole genome sequencing. We identified a novel pathogenic variant c.1675-1256_*115delinsGCAACATTTCGGCAACATTCCAACC in the *DARS2* gene. Three patients (patients 1, 2, and 4) had slowly progressive cerebellar ataxia, and two patients (patients 1 and 2) had spasticity. In addition, two patients (patients 2 and 4) showed signs of axonal neuropathy, such as decreased tendon reflexes and loss of distal sensitivity. Three patients (patients 1, 2, and 3) also had learning difficulties. It should be noted the persistent presence of characteristic changes in brain MRI in all patients, which emphasizes its importance as the main diagnostic tool for suspicion and subsequent confirmation of LBSL. Conclusions**:** We found a novel indel variant in the *DARS2* gene in four patients with LBSL and described their clinical and genetic characteristics. These results expand the mutational spectrum of LBSL and aim to improve the laboratory diagnosis of this form of leukodystrophy.

## 1. Background

Leukoencephalopathy with brainstem and spinal cord involvement and lactate elevation (LBSL; OMIM#611105) is an inherited disease caused by pathogenic biallelic variants in the gene *DARS2*, and was first described by Van der Knaap et al. [1]. The *DARS2* gene encodes a member of the class II aminoacyl-tRNA synthetase family on chromosome 1q25.1. It is a mitochondrial enzyme that specifically aminoacylates aspartyl-tRNA. In the case of LBSL, there is a reduction in the activity of the mitochondrial aspartyl-tRNA synthetase enzyme, leading to the impaired addition of aspartic acid to mitochondrial proteins [2]. Rumyantseva et al., 2020, showed that DARS2 depletion causes severe mitochondrial dysfunction concomitant with a massive loss of Purkinje cells (PCs) by the age of 15 weeks, thereby rapidly deteriorating motor skills [3]. Their findings conclusively showed that *DARS2* is indispensable for PC survival and highlighted the central role of neuroinflammation in DARS2-related PC degeneration. LBSL is characterized by slowly progressive pyramidal and cerebellar dysfunction, often with concomitant dorsal column dysfunction [1]. This disease typically manifests as a gradual decline in motor function, which commonly starts during childhood or adolescence but can occasionally occur in adulthood. The diagnosis of LBSL should be suspected in individuals with characteristic changes in the cerebral and cerebellar white matter and selective involvement of white matter tracts in the brainstem and spinal cord observed on MRI [1,2,4]. The mutational spectrum of *DARS2* includes 78 pathogenic variants registered in the Human Gene Mutation Database (HGMD, version 2022.1), 52% of which are missense substitutions. The ClinVar database (10 February 2024) includes 77 pathogenic variants, including large heterozygous deletions, which were established in microdeletion syndromes or other “non-LBSL” pathologies. Apparently, until now, there are only three gross deletions that have been described in LBSL patients: one of them is a 47 bp deletion in exon 7, and two others involve exons 12 and 15, respectively; the size of them was not specified in the publications [2,5,6]. Until today, there has been no evidence of gross deletions being repetitive mutations in LBSL. In almost all affected individuals, one pathogenic variant is present upstream of exon 3. The variant c.228-21_228-20delTTinsC is most often observed. In other affected individuals, nucleotide changes are seen in the same region within a stretch of ten or eleven C residues that lie ten nucleotides upstream of exon 3 [2]. Molecular genetic testing approaches can include a combination of gene-targeted testing (e.g., single-gene testing and multigene panels) and comprehensive genomic testing (e.g., exome sequencing, genome sequencing, and exome arrays). In this article, we present four cases where we found rare gross deletions believed to be a common variant for Russian patients with LBSL.

## 2. Materials and Methods

### 2.1. Editorial Policies and Ethical Considerations

This work was carried out in accordance with the Code of Ethics of the World Medical Association Declaration of Helsinki for experiments involving humans. This study was approved by the local ethics committee of the Federal State Budgetary Institution “Research Centre for Medical Genetics”. The approval number for this study is 2015–5/3 (dated 3 May 2015). Informed consent was obtained from all patients.

### 2.2. Patients

Four patients (2 females and 2 males) from four unrelated families were examined. The diagnosis of LBSL was verified by the genealogical analysis, clinical picture, brain MRI, and the results of the molecular and genetic analyses.

### 2.3. Molecular Genetic Methods

Genomic DNA was extracted from blood samples with the use of a QiaAMP DNA-mini kit (Qiagen, Germantown, MD, USA), following the manufacturer’s protocol.

Sanger sequencing was performed using an ABI Prism 3500XL (Thermo Fisher Scientific, Waltham, MA, USA), following the manufacturer’s protocol.

### 2.4. Searching for Common Mutations in the DARS2 Gene

The search for common pathogenic variants in the *DARS2* gene (e.g., c.228-21_228-20delinsC, c.455G>T (p.Cys152Phe), and c.492+2T>C) was carried out by automated Sanger sequencing, according to the manufacturer’s protocol, on the ABIPrism 3500xl device (Applied Biosystems, Waltham, MA, USA). Primers were designed according to the Refseq *DARS2* sequence NM_018122.5.

### 2.5. Target Gene Sequencing

*DARS2* whole gene sequencing was performed in all cases at the second line of the diagnostic protocol. Samples underwent target gene panel sequencing with the Ampliseq 59 genes panel, comprising major genes for inherited monogenic forms of leukodystrophies, including *DARS2*. Library preparation was made according to the manufacturer’s protocol. Sequencing was performed on Miseq (Illumina, San Diego, CA, USA).

Whole genome sequencing was performed with T-7 (MGI, Shenzhen, China) using PCR-Free sample preparation.

Bioinformatic pipeline: Sequence reads were aligned to the human reference genome hg19 using the Burrows–Wheeler Aligner (http://bio-bwa.sourceforge.net/ accessed on 10 Ferbuary 2024). Single-nucleotide variants and small insertions and deletions (indels) were called with the Strelka2 Small Variant Caller (https://github.com/Illumina/strelka accessed on 10 February 2024) and the Genome Analysis Toolkit v.4 (https://gatk.broadinstitute.org/ accessed on 10 February 2024). Structural variants were searched with Manta (https://github.com/Illumina/manta accessed on 10 February 2024) and Delly (https://github.com/dellytools/delly accessed on 10 February 2024). A manual search for discordant and split reads was performed using the IGV browser (https://www.igv.org/ accessed on 10 Februaru 2024). The reported variants were annotated with their genomic coordinates, allele frequency (gnomAD database, http://gnomad.broadinstitute.org/ accessed on 10 February 2024), functional consequence, and impact level on the gene product using SnpEff v5 (http://pcingola.github.io/SnpEff/ accessed on 10 February 2024). Variants were prioritized by the consensus score of the set of bioinformatic tools, which predict the pathogenicity of the variant and the deleterious effect on proteins (SIFT, SIFT4G, Polyphen2, MutationAssessor, FATHMM, PROVEAN, DEOGEN2, LRT, PrimateAI, MetaSVM, MetaLR, SpliceAI, MMsplice, SPiP, and Spidex). Data analysis was performed with an in-home NGS data–genome interface. All variants revealed by massive parallel sequencing were validated by Sanger sequencing in all patients and both of their parents. Variants were named according to the HGVS nomenclature and validated using VariantValidator (https://variantvalidator.org accessed on 10 February 2024).

### 2.6. Multiplex PCR for the Fast Detection of Novel Variants

**Figure 1:**
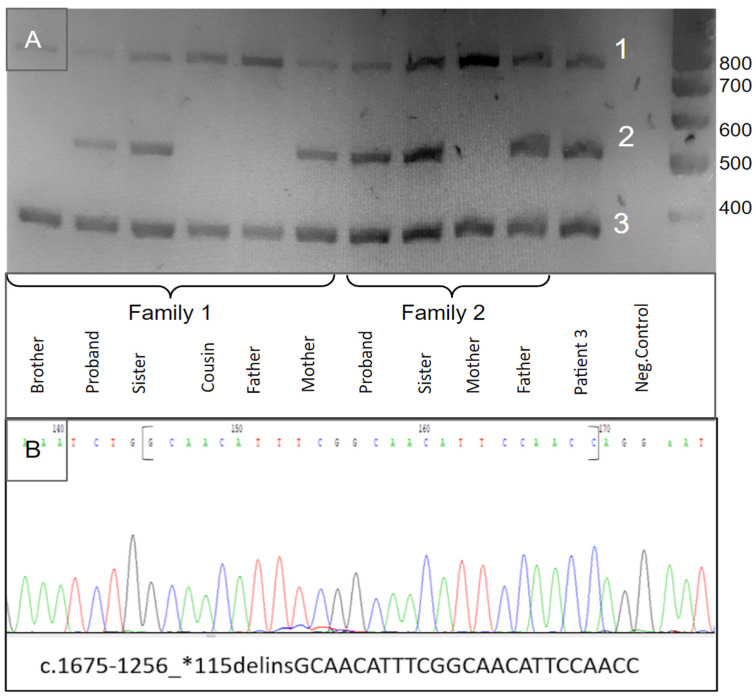
(**A**) Agarose gel detection of the novel indel variant. 1. PCR product for exon 17 and 3′ region detection—present in normal control and heterozygous carriers for the novel indel variant (forward primer of exon 17 and the reverse primer flanking the deletion—753 bp); 2. indel-specific PCR product—present only in samples with novel indel variant (flanking primers for the deleted region—516 bp); 3. PCR product for 17 exon detection—present in normal control and heterozygous carriers for the novel indel variant—386 bp. (**B**) PCR product 2 Sanger sequencing chromatogram with the novel indel variant.

The primer sequences for multiplex PCR were selected according to the reference sequence of the *DARS2* gene target regions (NM_018122.5) and included 2 pairs of primers flanking the deletion and primers for exon 17 of this gene, located inside this deletion (Table 1). The resulting PCR fragments were visualized using a 1% agarose gel. As a result, 3 fragments were identified: a deletion-specific fragment—516 bp, exon 17—a specific fragment—386 bp, and a cross fragment, including the forward primer for exon 17 and the reverse primer flanking the 3′ end of the deletion—753 bp (Figure 1 and Figure S1). 

## 3. Results and Discussion

The molecular diagnosis of LBSL is established through the identification of biallelic pathogenic variants in the *DARS2* gene, determined via molecular genetic testing in the proband exhibiting suggestive clinical findings. Our center pioneered LBSL molecular diagnosis 19 years ago, having successfully diagnosed over 250 patients from unrelated families to date, employing a standardized protocol encompassing both partial and whole gene sequencing.

Previously, all patients analyzed in our center demonstrated the presence of two pathogenic alleles. In the current study, patients 1 and 2 underwent testing following the same laboratory protocol for LBSL diagnostics. The initial step involved Sanger sequencing for frequent mutations, revealing one pathogenic allele (c.228-21_228-20delTTinsC) in a heterozygous state. However, the targeted gene panel analysis failed to identify the second mutant allele. Consequently, whole genome sequencing, focusing on intronic variants and structural rearrangements, was pursued.

In contrast, for patients 3 and 4 in this study, we deviated from the whole genome sequencing approach. Instead, a multiplex PCR system was employed to detect the gross indel variant previously identified in patients 1 and 2.

### 3.1. Clinical Cases

#### 3.1.1. Patient 1

A 12-year-old female patient, born to healthy, non-consanguineous parents, exhibited normal early motor development. However, from the age of 3 y., she underwent speech therapy for dysarthria. At 10 y., she began to complain of gait disturbance and walking clumsily, along with a decreased memory and attention span, leading to a decline in her school performance. Additionally, she presented with dysgraphia and decreased visual acuity. 

**Figure 2:**
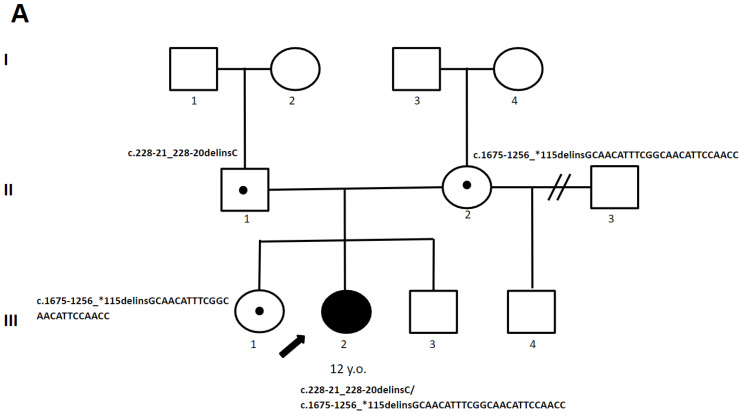

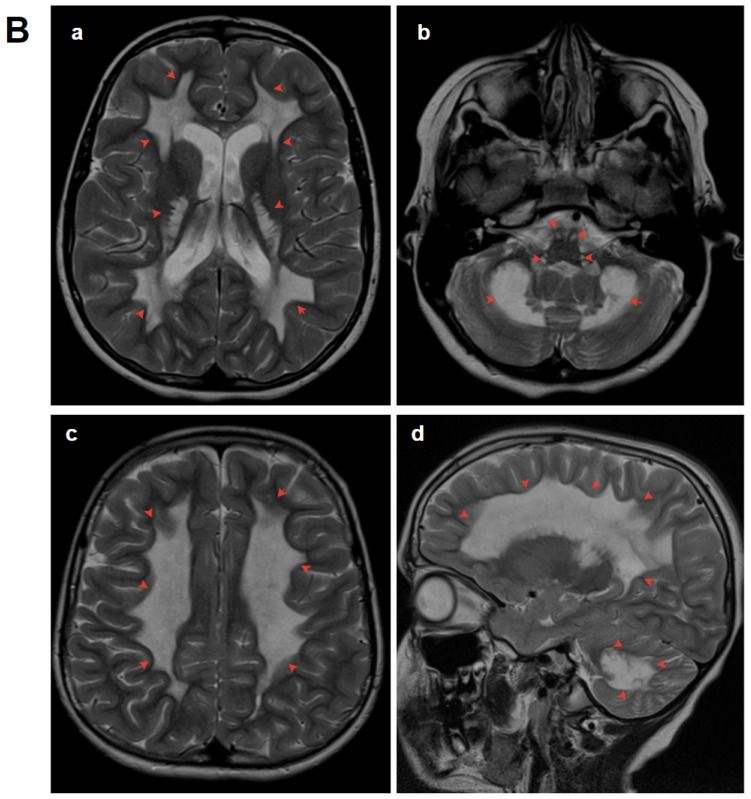
(**A**) The pedigree of patient 1. (**B**) MRI images of the brain of patient 1: (**a**,**b**,**d**) extensive, diffuse cerebral white matter abnormalities (red arrow); (**c**) selective involvement of the pyramidal tracts, superior and inferior cerebellar peduncles, and cerebellum (red arrow). areas I–III—generations; 1–4—the serial number of the family member; white circles—female gender; white squares—male sex; circle in subfigure—heterozygous carrier; black arrow—proband.

Upon her clinical examination at the age of 12 y., this patient displayed sensory ataxia, divergent strabismus, head and arm tremor, dysarthria, dysgraphia, cogwheel rigidity, hyperreflexia, clonus, Babinski reflex, and instability in the Romberg position. Her brain MRI (Figure 2) revealed specific signs of LBSL, including symmetrical leukoencephalopathy in the periventricular and deep white matter, as well as in the brainstem and cerebellum.

#### 3.1.2. Patient 2

A 14-year-old male patient, born to healthy, non-consanguineous parents, exhibited normal early motor development. At the age of 7 y., he was transferred to a remedial school and subsequently placed under the care of a psychiatrist due to exhibiting mild cognitive deficits. By the age of 8 years, he began experiencing a gait disturbance with slow progression. At 12 y., he reported headaches, leg pain, and a periodic loss of visual acuity.

**Figure 3:**
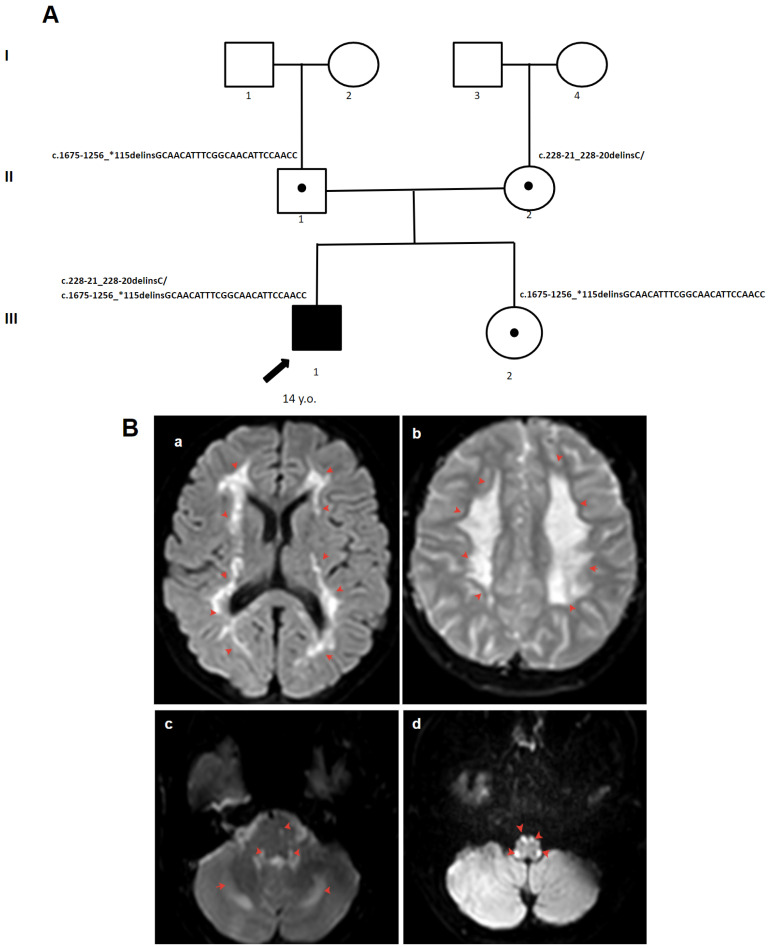
(**A**) The pedigree of patient 2. (**B**) MRI images of the brain of patient 2: (**a**,**b**) extensive cerebral white matter abnormalities (red arrow); (**c**,**d**) selective involvement of the pyramidal tracts, superior and inferior cerebellar peduncles, and cerebellum (red arrow). areas I–III—generations, 1–4—the serial number of the family member, white circles—female gender, white squares—male sex, circle in subfigure—heterozygous carrier, black arrow—proband.

His brain MRI (Figure 3) revealed diffuse signal elevation in T2WI in the corticospinal and sensory pathways of the brainstem and spinal cord, as well as in the cerebellum. Electroneuromyography (ENMG) demonstrated a moderate axonal-demyelinating polyneuropathy. An examination at 13 y. revealed a paretic gait with ataxia, generalized tremor, muscle atrophy in the distal parts of the leg, decreased tendon reflexes, and muscle strength in the legs (Grade 4), lower paraparesis, and ankle contractures.

#### 3.1.3. Patient 3

**Figure 4:**
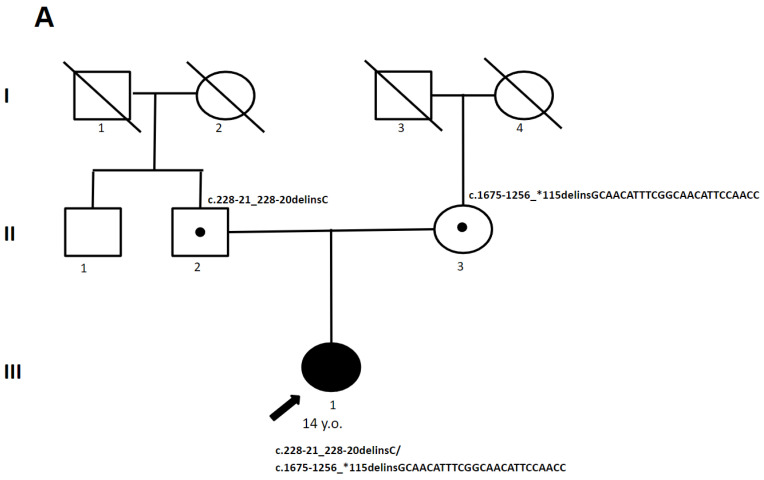

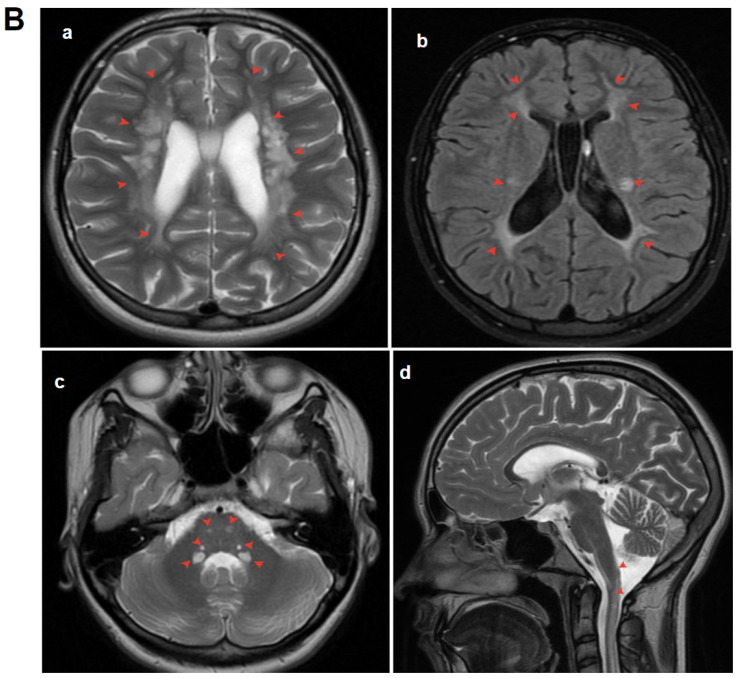
(**A**) The pedigree of patient 3. (**B**) MRI images of the brain of patient 3: (**a**,**b**) spotty cerebral white matter abnormalities (red arrow); (**c**) selective involvement of the pyramidal tracts and superior and inferior cerebellar peduncles (red arrow); (**d**) brainstem and spinal cord involvement (red arrow). areas I–III—generations, 1–4—the serial number of the family member, white circles—female gender, white squares—male sex, circle in subfigure—heterozygous carrier, black arrow—proband.

A 14-year-old female patient, born to healthy, non-consanguineous parents, exhibited signs of clumsiness in her gait from her moment of independent walking at 12 months. By the age of 7 y., she developed spastic paresis in her lower limbs, and by 13 y., pes cavus had formed. At the age of 13 y., a brain MRI was conducted, revealing multiple foci of altered white matter in the brain, brainstem, cerebellum, corpus callosum, and corticospinal tracts of the spinal cord (Figure 4). By the age of 14 y., she began to have learning problems.

A clinical examination at the age of 14 y. revealed horizontal nystagmus, distal weakness (Grade 4) and atrophy in her lower limbs, ankle joint contractures, pes cavus, intact tendon reflexes in her hands, heightened reflexes in her feet, Babinski sign, undisturbed sensitivity, and a paretic gait. 

#### 3.1.4. Patient 4

**Figure 5:**
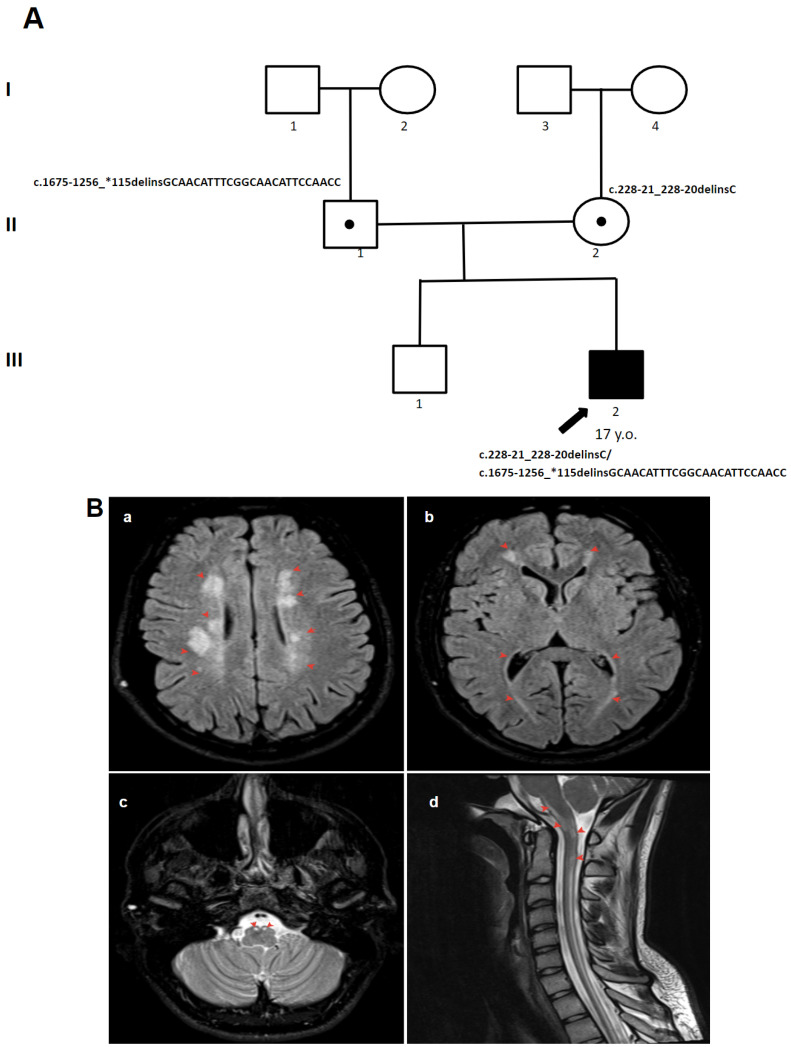
(**A**) The pedigree of patient 4. (**B**) MRI images of the brain of patient 4: (**a**,**b**) spotty cerebral white matter abnormalities (red arrow); (**c**) selective involvement of the pyramidal tracts and superior cerebellar peduncles (red arrow); (**d**) brainstem and spinal cord involvement (red arrow). areas I–III—generations, 1–4—the serial number of the family member, white circles—female gender, white squares—male sex, circle in subfigure—heterozygous carrier, black arrow—proband.

A 17-year-old male patient, born to healthy, non-consanguineous parents, demonstrated normal early motor development. Since the age of 12 y., he has been experiencing gait disturbances, characterized by an inability to stand on his heels and a steppage gait, along with leg weakness. These symptoms became more pronounced by the age of 15 y. Electroneuromyography (ENMG) revealed moderate manifestations of demyelinating motor–sensory neuropathy. A brain MRI showed multifocal lesions in the brain, brainstem, and spinal cord (Figure 5), raising suspicion of multiple sclerosis; however, this diagnosis was subsequently ruled out.

During his examination at the age of 17 y., this patient exhibited horizontal nystagmus, distal weakness in his lower limbs (Grade 4 for hip flexion and foot dorsiflexion) with associated hypotrophy, decreased tendon reflexes in his hands and increased reflexes in his legs, ankle contractures, Babinski sign, decreased deep sensation, instability in the Romberg’s position, postural tremor, and sensory ataxia.

### 3.2. Molecular Findings

**Figure 6:**
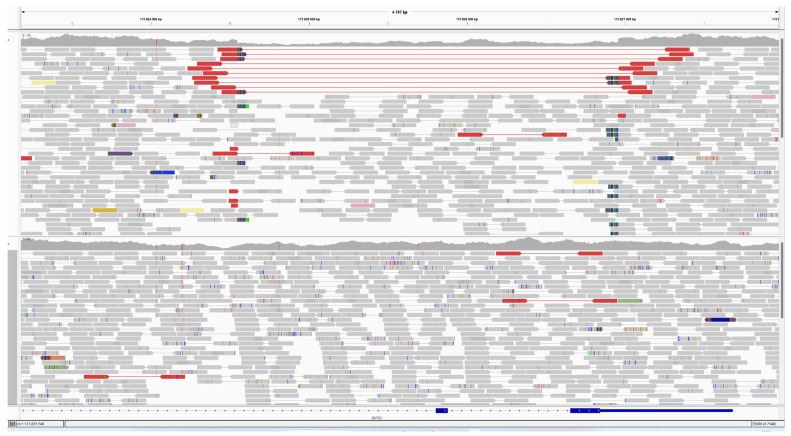
IGV browser view depicting a part of the DARS2 gene. The upper track represents patient 2, while the lower track represents a normal control sample. Discordant and split reads observed in the upper track strongly indicate the boundaries of the deletion.

Whole genome sequencing revealed a group of paired discordant and split reads in the *DARS2* gene. After grouping the reads in the IGV browser (Figure 6), a large heterozygous deletion was suspected. Split reads showed the breakpoints, accompanied by a sharp coverage drop. This deletion spans 2411 bp (Chr1:173824548-173826958 (hg19)) and includes exons 16 and 17 (the two last exons of the *DARS2* gene). To classify new CNVs, we employed ACMG and ClinGen guidelines [7]. The minimal score for pathogenic CNVs is 0.99 and a novel deletion accounts for 2.2, so they could definitely be classified as pathogenic. This variant was submitted to the ClinVar database (SCV004698191). Sanger sequencing with flanked primers confirms and specifies the rearrangement as a deletion with insertion: c.1675-1256_*115delinsGCAACATTTCGGCAACATTCCAACC. Sanger sequencing in the probands and parents confirmed autosomal recessive inheritance (parents occurred to be heterozygous for one of the identified variants—frequent pathogenic variant c.228-21_228-20delTTinsC or novel indel c.1675-1256_*115delinsGCAACATTTCGGCAACATTCCAACC).

**Figure 7:**
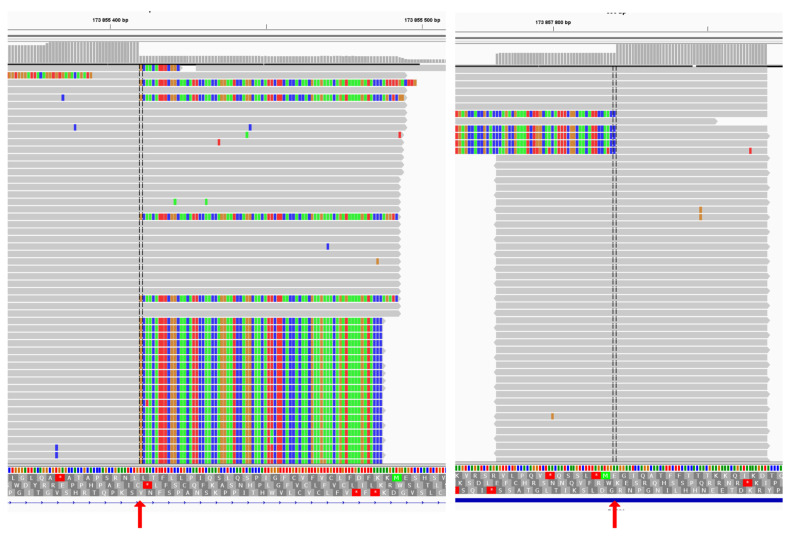
Left panel—5′ end of the novel indel visualizing via the IGV browser at the target genes panel. Right panel—3′ end of the novel delins. Red arrows point on the break-point of the indel showing with the split reads. The gray upper track of coverage also has a sharp drop in the breakpoint.

After revealing two patients with the compound heterozygous novel indel variant, we developed a fast multiple-PCR test for accurate detection of this variant at the first line of the laboratory diagnostic of LBSL. Using this test, we revealed two other unrelated patients with LBSL having this novel variant in a compound state with the frequently pathogenic c.228-21_228-20delTTinsC variant (patients 3 and 4). For further improvement of the diagnostic approaches, we developed a new target gene panel for monogenic forms of leukodystrophies, including c.1675-1256_*115delinsGCAACATTTCGGCAACATTCCAACC *DARS2* variant identification (Figure 7). Using this panel, we confirmed the same genomic localization of the indel’s breakpoints in patients 3 and 4.

In the gnomAD v4.1.0 database, we identified eight different large deletions ranging from 559 bp to 4.78 kb within the *DARS2* gene, all predicted to result in loss-of-function consequences. Unfortunately, there is no whole genome data specific to the population of the Russian Federation, hindering our ability to estimate the prevalence of the identified indel carriers within our population. However, we have found a common *DARS2* pathogenic large indel that has spread to the territory of Russia. It is interesting to note that in earlier times, large deletions and rearrangements in the *DARS2* gene were a very rare course of LBSL cases from around the world, but now we can speculate that in Russia, it could be more undiagnosed patients in retrospective, in those with the absence of the second mutant allele after common diagnostic tests, including whole exome sequencing.

## 4. Conclusions

The application of whole genome sequencing (WGS) technology has enabled the identification of a novel indel variant in the *DARS2* gene across four unrelated patients diagnosed with LBSL in Russia. This discovery not only expands the mutational spectrum associated with LBSL but also significantly contributes to enhancing the precision of diagnostic procedures. Despite exhibiting normal motor development initially, all patients experienced a gradual decline in motor skills during childhood or early adolescence.

In three patients (patients 1, 2, and 4), a slowly progressive cerebellar ataxia was observed, while two patients (patients 1 and 2) manifested spasticity. Additionally, signs of axonal neuropathy, including decreased tendon reflexes and loss of distal sensation, were identified in two patients (patients 2 and 4). Learning difficulties were reported in three patients (patients 1, 2, and 3). Notably, characteristic changes in brain MRIs were detected in all patients, serving as a crucial indicator for the suspicion and subsequent confirmation of LBSL.

In summary, the utilization of WGS technology has not only unveiled a new indel variant in the *DARS2* gene but has also deepened our understanding of the clinical manifestations associated with LBSL. This breakthrough aids in expanding diagnostic capabilities and underscores the importance of genetic analysis in unraveling the complexities of neurodegenerative disorders.

## Figures and Tables

**Table 1:** Clinical characterization of patients with LBSL reported in the present study.

Patient Number	1	2	3	4
**Gender**	f.	m.	f.	m.
**Age at the last observation (years)/** **age of onset**	12 y./ 10 y.	14 y./ 3 y.	14 y./ 1 y.	17 y./ 12 y.
**Early development**	Normal	Normal	Normal	Normal
**First symptoms**	Gait disturbances	Gait disturbances	Gait disturbances	Gait disturbances
**Headache**	−	+	−	−
**Cognitive decline**	Mild	Mild	−	−
**Nystagmus**	−	−	−	Horizontal nystagmus
**Decreased visual acuity**	+	+	−	−
**Dysarthria**	+	−	−	−
**Dysgraphia**	+	−	−	−
**Peripheral axonal neuropathy**	−	+	−	+
**Distal weakness**	−	−	−	+
**Leg pain**	+	+	−	−
**Muscle atrophy**	−	Lower limb atrophy	Lower limb atrophy	+
**Spasticity**	+	+	−	−
**Tremor**	Hand tremor	Generalized tremor	−	Hand tremor
**Tendon reflexes**	Hyperreflexia	Tendon reflexes are decreased	Lower limb hyperreflexia	Tendon reflexes in the hand are decreased; lower limb hyperreflexia
**Extensor plantar responses**	Babinski reflex and clonus	−	Babinski reflex and clonus	Babinski reflex
**Pes cavus**	−	+	−	+
**Joint contractures**	−	Ankle contractures	Ankle contractures	Ankle contractures
**Ataxia**	+	+	−	+
**Gait**	Sensory ataxia	Paretic gait with ataxia	Paretic gait	Sensory ataxia

## Data Availability

Dataset available on request from the authors.

## Supplementary Materials

The following supporting information can be downloaded at: https://www.mdpi.com/article/10.3390/genes15050615/s1, Figure S1: Primer’s sequences for multiplex analysis for novel indel variant in the DARS2 gene.

## Abbreviations

LBSL	Leukoencephalopathy with brainstem and spinal cord involvement and lactate elevation
WMH	White matter hyperintensities
MRI	Magnetic resonance imaging
T1WI	T1-weighted images
ENMG	Electroneuromyography
