# Supplementary material for: Identification of a Novel Indel Variant in the DARS2 Gene in Russian Patients with Leukoencephalopathy with Brainstem and Spinal Cord Involvement and Lactate Elevation
Source: Genes (Basel). 2024 May 11;15(5):615. doi: 10.3390/genes15050615 (PMC11121371; doi:10.3390/genes15050615)
Supplement: Supplementary file 1 [file genes-15-00615-s001.zip › genes-2990633-supplementary.pdf]

Target	Location	Forward	Reverse
DARS2	flanking the indel c.1675-1256_*115delinsGCAACATTTCGGCAACATTCCAACC	5’ GATTACACGTGCGCACTAACAC - 3	5’ GATTCTCTTGATGAGGATGATGAAG-3’
DARS2	17 ex	5’ GGAACGATGGTTGGCTACAG-3’	5’-GACTTTAGCCTTGGGGAAGC-5’

Suppl. Figure 1. Primer’s sequences for multiplex analysis for novel indel variant in the *DARS2* gene.
